# Patients’ perspectives on, experience with and concerns about crohn's disease: insights from Chinese social media

**DOI:** 10.1186/s12876-023-02747-x

**Published:** 2023-04-04

**Authors:** Shaopeng Sun, Yunhong Hu, Heng Li, Jiajia Chen, Yijie Lou, Chunyan Weng, Lixia Chen, Bin Lv

**Affiliations:** 1 The First Clinical Medical College, Zhejiang Chinese Medical University, Hangzhou, China; 2 Department of Anesthesiology, The First Affiliated Hospital of Zhejiang Chinese Medical University (Zhejiang Provincial Hospital of Chinese Medicine), Hangzhou, China; 3 Nursing College of Zhejiang Chinese Medical University, Hangzhou, China; 4 Department of Gastroenterology, The First Affiliated Hospital of Zhejiang Chinese Medical University (Zhejiang Provincial Hospital of Chinese Medicine), Hangzhou, China

**Keywords:** Crohn's disease, Qualitative study, Latent Dirichlet allocation, Grounded theory, Inflammatory bowel disease, Social media

## Abstract

**Aim:**

The aim of this study was to explore the experience and perceptions of patients with Crohn’s disease in China.

**Methods:**

Data mining was used to investigate posts in Crohn’s disease online medical communities. The data were collected through the crawler code, and latent Dirichlet allocation (LDA) and grounded theory were used to mine the theme features after data cleaning.

**Results:**

In analyzing the topic characteristics of online posts, LDA divided 6757 posts into 15 topics on four aspects: seeking disease information, making decisions on medication use, psychological burden, and communicating about diet and nutrition.

**Conclusion:**

Overall, social media is patient-centric and helps us better understand the experiences and perceptions of patients. This study can help medical staff predict the thoughts and concerns of Crohn's disease patients during the treatment process, facilitate doctor-patient communication, and assist in the formulation of medical policies.

## Core Tips

### What is already known?

Patients with Crohn's disease face many difficulties and challenges, especially in the context of Chinese culture, such as poor quality of life, lack of health education and social support, etc. in addition, there are few patient-centered studies.

### What is new here?

We collect extensive, high-quality and authentic patient experiences through social media to fully understand the perspectives, experiences and concerns of Chinese Crohn's disease patients.

### How can this study help patient care?

This study could help medical staff predict the thoughts and concerns of Crohn's disease patients during treatment, facilitate doctor-patient communication, and help shape healthcare policy.

## Introduction

Crohn's disease is a subtype of inflammatory bowel disease (IBD) characterized by a chronic inflammatory disorder of the gastrointestinal tract that requires lifelong medical treatment [1]. In the past, IBD has been considered a disease of the Western world; however, data from the last decade show a rising incidence in newly industrialized countries in Asia [2]. The incidence and prevalence of IBD are increasing rapidly, and it is no longer rare in China [3]. Considering the large population of China, it is predicted that the total number of IBD patients in China may exceed that in North America in the near future [4]. This evidence indicates that China will soon have a huge number of IBD patients, which will inevitably bring enormous challenges to the health system.

In the past, qualitative studies, such as questionnaire surveys and semistructured interviews, were used for patients with Crohn’s disease [5, 6]. One qualitative study explored the experience of twenty-five participants with IBD and moderate-to-severe symptoms and suggested that psychotherapists working with IBD need advanced skills to gain a clear understanding of the different processes impacting patients’ mood and levels of anxiety [6]. Another qualitative study reported on the challenges of living with and managing IBD and found that physical symptoms were the greatest challenges, negatively affecting patients’ psychological and social well-being and reducing their quality of life [7]. These qualitative studies focused on quality of life and mental health-specific interviews [6, 8]. However, such studies were designed and guided by researchers rather than being the direct perspective of patients. Most studies did not distinguish between subtypes of IBD: ulcerative colitis and Crohn's disease.

With the development of the internet, patients have gradually come to rely on it. An increasing number of patients tend to search online for relevant information before seeing a doctor or obtain medical services through the internet [9]. A study in the US found that 59% of users search for health information on the internet [10]. In addition, social media plays an important role for many people, including adolescents [11], pregnant women [12], and college students [13], especially in the context of COVID-19 [14]. Gabriela Gonzalez et al. [10] assessed women's knowledge, patient experience, and treatment decision making regarding overactive bladder using social media posts. These results have been of great help in clinical and nursing practices.

Social media provides us with an angle to better understand patients, especially in China, which has 800 million internet users. To gain a better understanding of the knowledge, perceptions and experience of Crohn's disease patients in Chinese online networks, we collected Crohn's disease-related posts from Chinese social media and explored the experience, perceptions, needs and difficulties of patients.

## Materials and methods

### Study design

Baidu Tieba (https://tieba.baidu.com/) is the largest Chinese online community; it consists of a variety of subcommunities on different topics and gathers massive user groups with different interests. As of May 20, 2022, the Crohn's disease bar (https://tieba.baidu.com/f?ie=utf-8&kw=%E5%85%8B%E7%BD%97%E6%81%A9) had more than 10,000 followers and more than 150,000 posts and replies. The massive amount of user data generated in this open community is of great significance for analyzing Crohn's disease patients’ experience and perceptions.

**Fig. 1:**
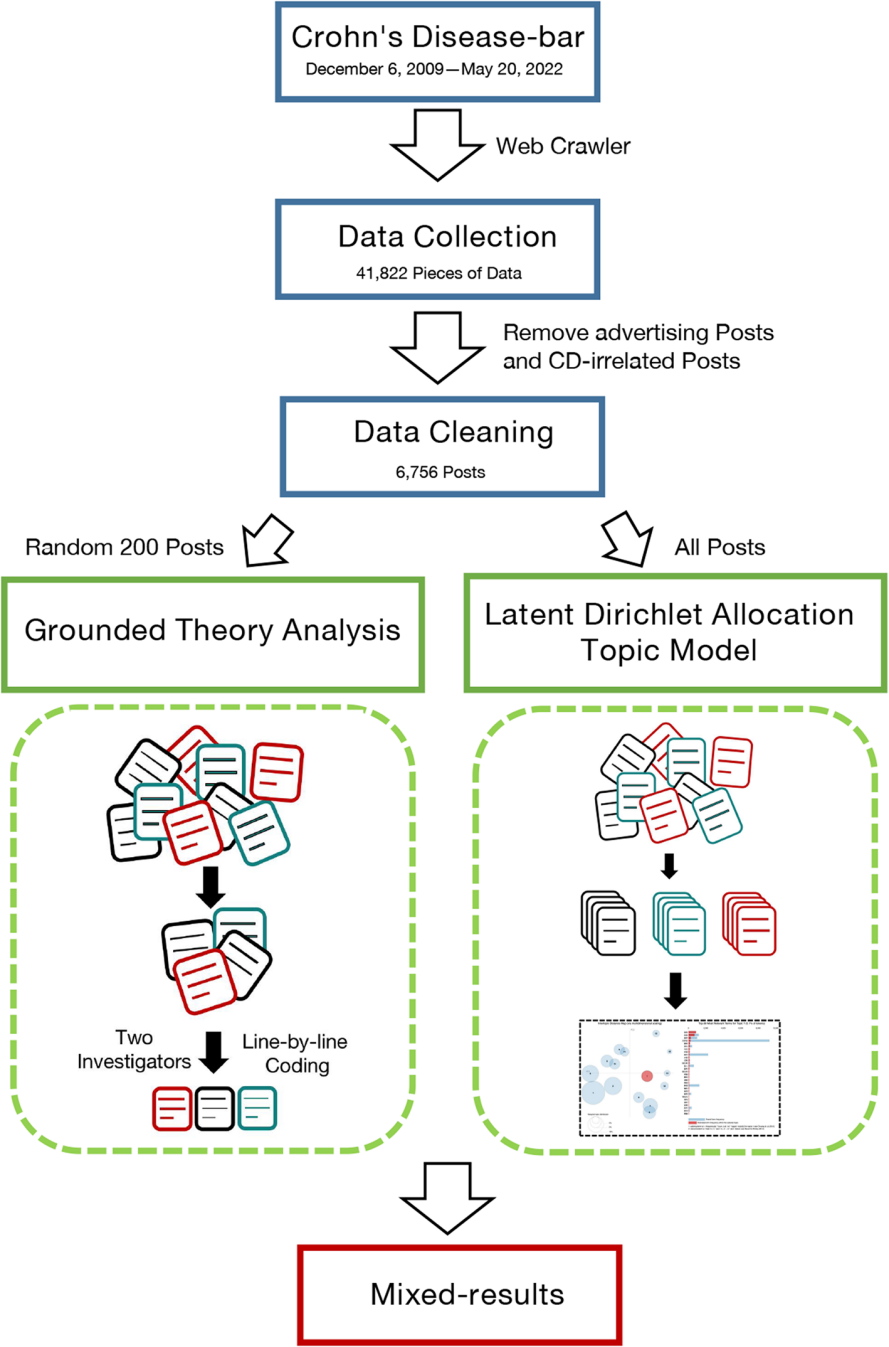
Flow chart of the study

In this study, we collected 12 years of posts from the Crohn's disease bar through data mining. After data cleaning, the latent Dirichlet allocation (LDA) topic model and grounded theory were used to explore the theme and understand the experience and perceptions of Crohn's disease patients based on the perspective of social media. The workflow is presented in Fig. 1.

### Data collection and cleaning

All user posts (including posts, comments and replies) were written in Chinese and published online between December 6, 2009, and May 20, 2022.

By using the ‘request’ and ‘selenium’ packages and fast web crawling based on Python, we extracted the required data from web pages. A total of 41,822 pieces of raw data were collected. Subsequently, each post was combined with corresponding comments and replies into a single document for further analysis.

We removed blank lines, duplicate text, and special symbols, numbers and letters from the text, further eliminated text with a string length of less than 10, and retained only Chinese strings. Additionally, we manually reviewed advertised posts and posts unrelated to Crohn's disease. Finally, a total of 6757 posts were used for further analysis.

### Qualitative methods: grounded theory

Once the posts were identified, we randomly selected 200 posts and used grounded theory methods for analysis, as described by Charmaz [15]. This number was chosen to achieve thematic saturation, meaning the point at which themes begin to be repeated.

Grounded theory is an iterative, hypothesis-generating approach that emphasizes the generalization or emergence of information from data to establish a theory or model [16, 17]. Two investigators individually coded line-by-line to identify prominent issues and concepts. Next, we reviewed the codes and incorporated them into coding categories, which are groups of similar concepts and themes. Data analysis was completed by NVivo12 plus software.

### Quantitative methods: LDA topic model

In addition to qualitative analysis, we quantitatively analyzed all posts using the LDA probabilistic topic model [18]. LDA assumes that documents are generated based on a certain number of topics and that a given number of topics can be extracted from a corpus containing a certain number of documents [19]. It is an excellent probabilistic model that performs well in topic modeling and has been widely applied in research [20].

In this research, we applied LDA to extract a certain number of topics from the cleaned dataset. To determine the number of topics, the coherence scores were taken into consideration [18]. Topic coherence is the most effective method of measuring the quality of topics and one of the important techniques used to estimate the number of topics. It measures the sentence similarity of each topic in the dataset, and higher scores represent the most appropriate number of model topics [21]. After the optimal model is determined, another Python package, ‘pyLDAvis’, was adopted to visualize the topic extraction results. We could then view the topic distance and overlap in a two-dimensional space. The size of different circles represents the topic distribution.

## Results

### LDA topic model analysis

**Fig. 2:**
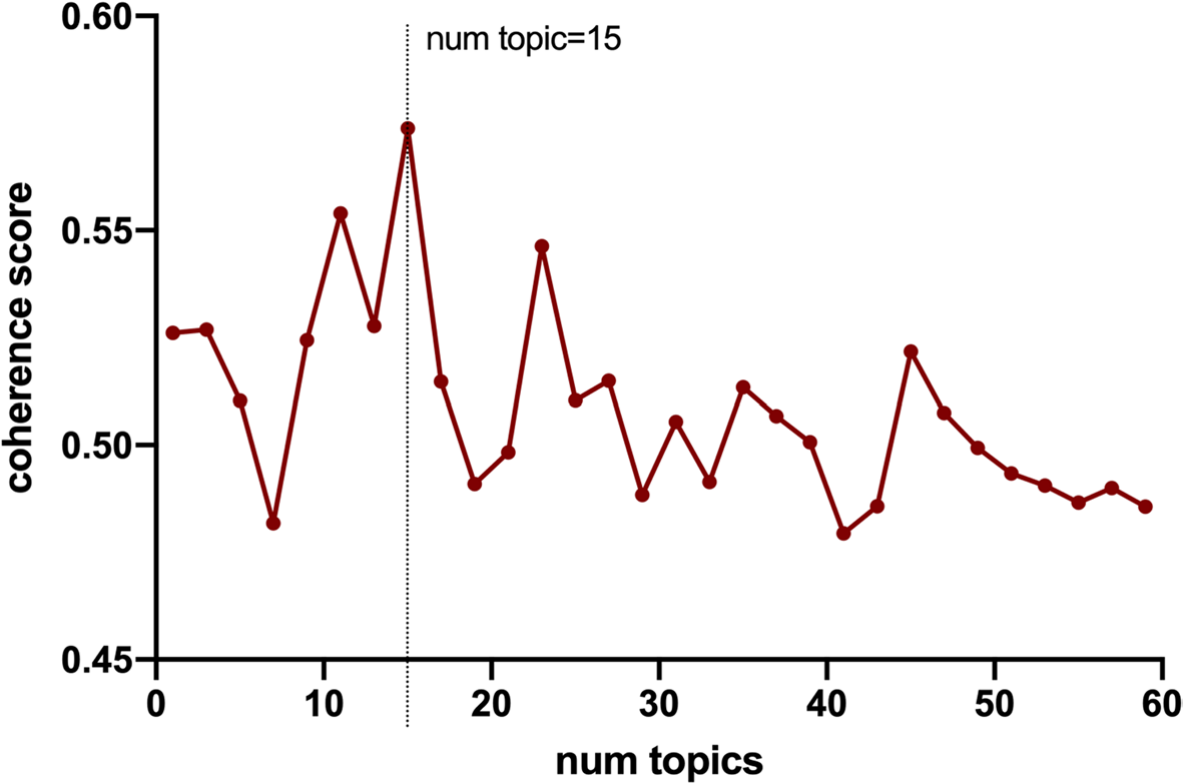
Coherence scores for different numbers of topics. The number of topics with the highest score is 15

**Fig. 3:**
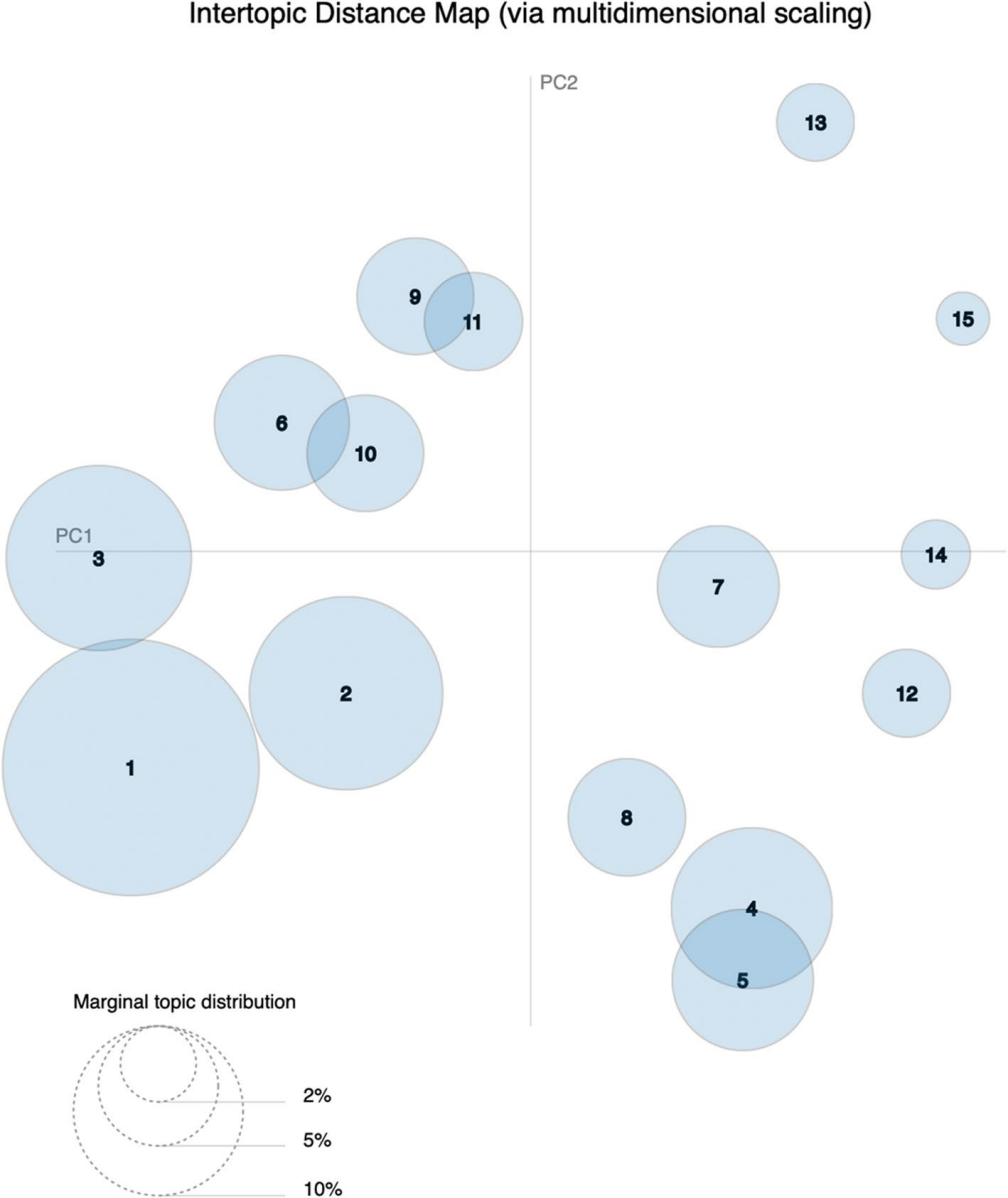
Intertopic distance map

We studied 6757 high-quality posts from Chinese social media that focused on Crohn's disease. Consistency scores were calculated from 1 to 60 topics to obtain the optimal number of topics (Fig. 2), and a total of 15 topics were found (Fig. 3).

Table 1 shows the representative term cluster with the each term’s prevalence and assigned topic. The majority of posts (22.8%) focused on discussions surrounding diagnosis and symptoms, and 13% talked about the impact of the disease on individuals and family. Some people (11.9%) expressed concern about complications and surgery, and 9% of the posts expressed expectations for new therapies. Among the patients, 6.9% wanted to explore the etiology and mechanism of the disease, 6.3% thought they lacked professional treatment support, and 5.1% were confused about diet and nutrition. Traditional Chinese medicine (TCM) (4.8%), biological agents (4.7%) and traditional therapeutic drugs (4.7%) were the most discussed drugs. Health insurance policies (3.3%), medication experience (2.7%) and possible drug side effects (2.1%) were also concerns for patients with Crohn's disease. In addition, financial situation (1.6%) and social impact (1%) were among their burdens.

**Table 1:** Representative term cluster with prevalence and assigned topic

Representative term cluster	Prevalence	Topics
Crohn's disease, colonoscopy, symptom, ulcer, abdominal pain, hematochezia, hospital, pathology, inflammation, gastroscope, large intestine, consult	22.8%	Diagnosis and symptoms
Mood, mentality, children, patient, parent, pain, stress, friend, love, wife, family, help, life, future, anxious, sleep, go home, husband, hope, daughter, time, collapse	13%	Individuals and family
Anal fistula, abscess, wound, operation, intestinal obstruction, nutrient solution, nasal feeding, hospitalization, complication, anus, arthritis, narrow, fibrosis	11.9%	Complications and surgery
Research, fecal bacteria transplantation, superoxide, activity, cytokine, clinical experiment, cell, news immune system, gene technology, stem cells, placebo, news	9%	New therapies
Pathogeny, sensitive, born, susceptibility, erosion, Pathology, histological examination, infect, alcoholism, habit, environment, food, granuloma	6.9%	Etiology and mechanism
Hospital, doctor, Beijing, Shanghai, expert, professor, Peking Union Medical College, Sun Yat-sen university, SIR RUN RUN SHAW hospital, Zhejiang, Zhejiang University, General Hospital of Eastern Theater Command, Nanjing, Wuhan, West China Medical Center, XIANGYA Hospital	6.3%	Professional treatment support
Food, diet, vegetables, nutrition, digestion, milk, vitamin, noodle, protein, fruit, egg, pepper, drinks, greasy, rice, seafood, fried, meat, bread, fluid	5.1%	Diet and nutrition
Traditional Chinese medical science, traditional Chinese medicine, meridians, Qi and blood, body fluid, spleen deficiency, *Codonopsis pilosula*, prescription, traditional Chinese medicine hospital, Chinese herbal enema	4.8%	Traditional Chinese medicine treatment
Infliximab, curative effect, Adalimumab, ustekinumab, biological agent, medication regimen, price, cost, injection, antitumor necrosis factor, antibody	4.7%	Biological agent
Hormone, Mesalazine, azathioprine, prednisone, white blood cell, take medicine, Tuberculosis, positive, drug addict	4.7%	Traditional therapeutic drugs
Medical insurance, reimbursement, discount, country, import, on the market, National Healthcare Security Administration, offsite medical treatment, bill service, Payment of hospitalization fee	3.3%	Health insurance policy
Side effect, allergy, pain, invalid, drug resistance, hemorrhage, innutrition, recrudescence, progress, feel, throat, occult blood, occult blood,	2.7%	Side effect
Taste, quality guarantee period, experience, curative effect, valid, insist, useless, share, attention, advice, taboos,	2.1%	Medication experience
Expensive, selling drugs, at one’s own expense, cost, countryside, money, exchange, amount of money, cheap, fundraising, poor, economic, waste, low price, sell	1.6%	Financial situation
Cause, work, government officials, student, obesity, appearance, university social intercourse, society, community, inferiority, trouble, interpersonal relationship, estranged, high school	1%	Social impact

### Grounded theory

We identified nine preliminary topics related to the experience and perceptions of Crohn's disease patients during data analysis using grounded theory: (i) seeking disease information, (ii) improving quality of life, (iii) mental burden, (iv) medications and treatment modalities, (v) diet and nutrition, (vi) disease progression, (vii) economic burden, (viii) peer support, and (ix) IBD expert panel support.

Most of the topics encoded by grounded theory were established in LDA topic modeling; however, an additional important topic was found in our coding analysis: peer support. Peer support was usually in the posts that shared patient experience and treatment experience. Patients with Crohn's disease can be encouraged to adhere to treatment (e.g., “I would like to share my treatment experience and hope to help you”). In addition, peer support also appeared in depressed posts for which encouragement, support and suggestions usually appeared in replies (e.g., “Cheer yourself up, you will recover”).

### Results of mixed methods

**Fig. 4:**
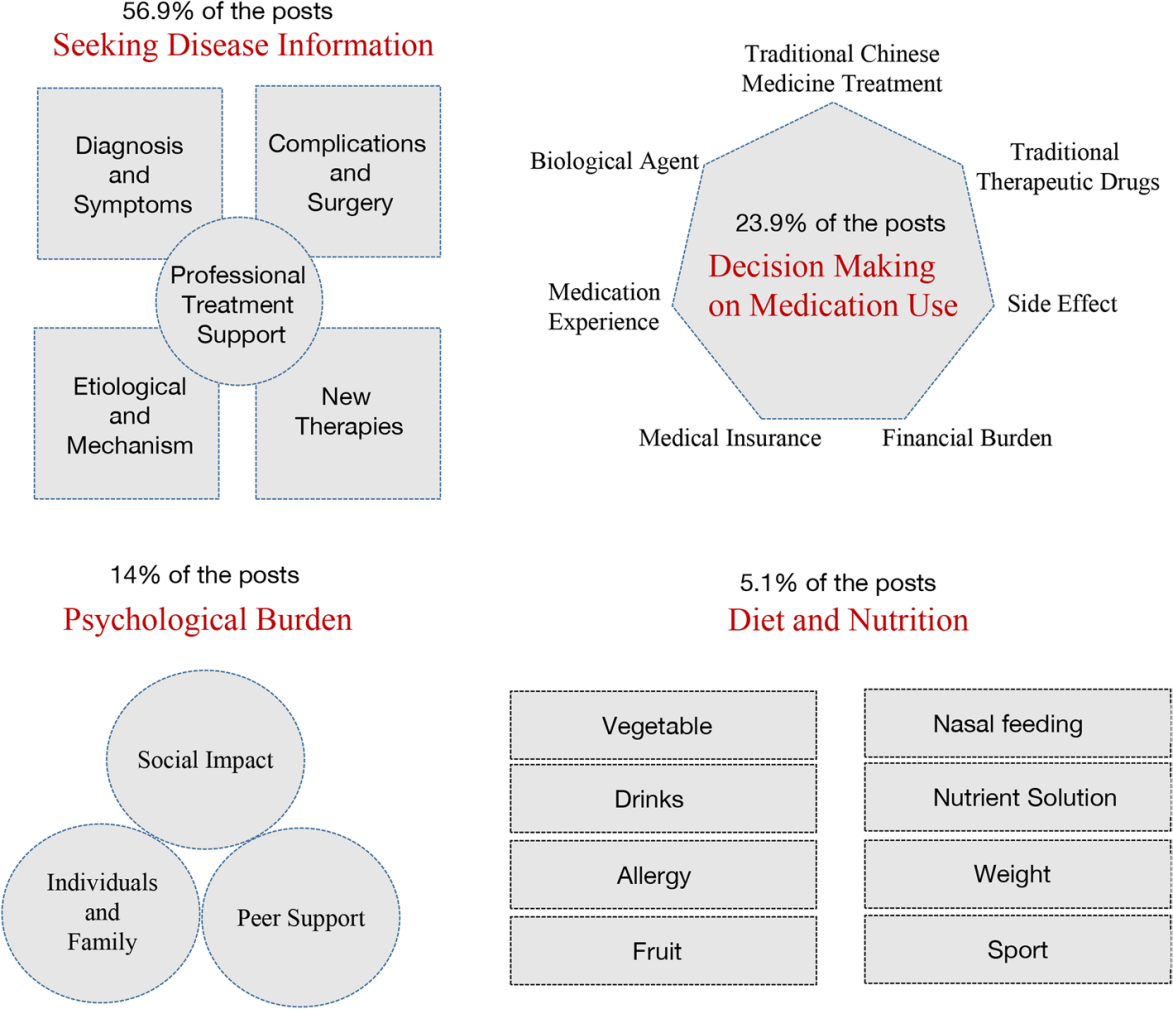
Themes identified by mixed methods

Topics were generated by LDA and compared to the themes identified in the grounded theory analysis. We identified five themes with several subthemes, which are summarized in Fig. 4.

### Results during the COVID-19 pandemic

The LDA model did not generate a topic related to the COVID-19 pandemic. As a result, we manually retrieved relevant posts about the COVID-19 pandemic from December 1, 2019 to May 20, 2022. The search terms included 'COVID-19', 'infection', 'pneumonia', 'epidemic situation' and 'epidemic prevention' resulting in 54 high-quality posts.

Consonant with a grounded theory approach, data were coded and analytic induction was used to identify key themes. Four main themes were identified: (i) Preventive and protective measures (e.g., “Is the resistance to the COVID-19 worse for Crohn’s disease patients than it is for ordinary people?”), (ii) COVID-19 vaccination decision (e.g., “Are there any patients who have been vaccinated with biological agents and with the COVID-19 vaccine? Can we get vaccinated?”), (iii) Drug use after COVID-19 infection (e.g., “Can biological agents be used when infected with COVID-19?”), (iv) Concerns about the shortage of medical resources (e.g., “For the sake of epidemic safety, the hospital temporarily closed the biological reagent injection room.”).

## Discussion

Health and disease belong to the category of biomedicine but also involve social, political and economic dimensions [22]. Our research summarized posts in the Crohn’s disease bar over the previous 12 years and conducted a comprehensive analysis through two methods: grounded theory and the LAD topic model. In contrast to previous interview studies with small sample sizes, this study analyzed Chinese patients with Crohn's disease from the perspective of social media. An enormous and comprehensive dataset identified the prominent concerns of patients with Crohn's disease. This study provides novel insight into patients’ experiences with Crohn's disease and identifies actionable needs that may improve their quality of life.

The need for shared decision making in IBD has become a consensus among IBD physicians worldwide [23]. However, there is a large gap between doctors and patients in their knowledge and information about diseases. Apart from online consultation, Crohn's disease patients seem to have few ways to acquire such knowledge. Our study confirmed that 56.9% of Crohn's disease patients urgently want more information about the disease, including diagnosis, symptoms, pathology, complications, surgery, new therapies and professional treatment support. This need is more urgent for patients who have symptoms but have not been diagnosed or have difficulty in diagnosis. Consulting patients who have been diagnosed is the most common method. For instance, some people described their symptoms and indicated that they have undergone some physical examination (but had not received feedback). They were very afraid, especially after they searched for information about Crohn's disease on the internet. Some low-quality answers often have this effect. Patients' consultation of social media negatively influenced the diagnostic and therapeutic decisions. For example, some posts mentioned that the anesthetics used in painless enteroscopies would damage the memories. This misunderstanding has always existed in China and has negatively affected many people, causing them to suffer needlessly by refusing to undergo anesthesia during an enteroscopy. In addition, some posts mentioned consulting experts and hospitals with experience in treating Crohn's disease. Some patients find their disease progressing because of misdiagnosis, untimely treatment and nonstandard treatment.

Encouragingly, many Crohn's disease patients in China had shared their experiences through video platforms, such as the Chinese Tik Tok and Bilibili video platforms; some self-media medical accounts also disseminated Crohn's disease knowledge through popular science articles and videos. In recent years, the Chinese Crohn's and Colitis Foundation (CCCF) [24], a large nongovernmental public welfare organization that is committed to improving the quality of life of IBD patients in China by providing social resources, has made great contributions through patient education, public awareness, helping to train IBD specialists, etc. In addition, an increasing number of hospitals are actively training IBD specialists and establishing multidisciplinary teams (MDTs) [25].

Pharmacotherapy is the first step in treating IBD [26]. The main medicine for the management of Crohn's disease works by suppressing an overly active intestinal immune system [26]. Several monoclonal antibodies have been approved for the treatment of Crohn's disease, such as anti-TNF agents [27] and anti-integrin agents. In addition, the use of these biologics in combination with a conventional immunosuppressant may yield superior outcomes and improve the durability of therapy [28, 29]. In our analyses, we also found that more than 1/4 of the posts discussed the drug treatment of Crohn's disease, and seven subthemes were mentioned repeatedly. Biological agents, traditional therapeutic drugs, hormones and TCM were the drugs of most concern to patients. Other patients' medication experience (efficacy and recommendations) and side effects also affected their medication decisions. Medical insurance policies and their own financial situation restricted them from choosing better drugs.

Interestingly, Chinese patients with Crohn's disease were willing to use TCM for treatment, especially when biological agents failed. Cai et al. used semistructured interviews with 14 IBD patients using TCM and explored their reasons and experience of decision-making [30]. The results showed that patients initiated TCM treatment due to the unsatisfactory effects of other therapies. TCM has been widely used to treat IBD in China; however, systematically determining its efficacy in IBD treatment is still challenging [31].

The benefits and risks of treatment are the primary consideration of doctors, which also places huge decision-making pressure on patients. A previous network survey on Crohn's disease patients found that the acceptable risk of drug treatment for Crohn's disease patients depends on the severity of the disease and the expected benefits. Especially for patients with mild to moderate cases, the acceptance of risk is low [23]. Another study showed that when Crohn's disease patients decide to use biological agents, they will comprehensively consider the safety and efficacy of those agents. Among them, safety accounted for 54% of the attention and the efficacy only 28% [32]. Our results are consistent with these findings. Many patients posted questions about the side effects of drugs and expressed their concerns. The experience of other patients often affected their decisions about medicine.

In our study, financial burden was another important influencing factor of drug use decisions, which was closely related to medical insurance policies. A retrospective study of the medical expenses of Crohn's disease patients based on a medical insurance database in the United States showed that the average lifetime medical cost of Crohn's disease patients was $416,352 higher than that of non-IBD patients, and the younger the diagnosis age was, the higher the lifelong medical expenses [33]. One two-center study on the treatment costs of Chinese Crohn's disease patients found that by 2011, the average hospitalization expenses of Crohn's disease patients had risen to CNY25504.21, an increase of 237% over 10 years ago [34]. The medical insurance policies of different provinces in China differ, and different reimbursement policies are formulated according to the severity of Crohn's disease, which also leads to different drug choices for patients.

Depression and anxiety are common in patients with IBD and are increasingly being recognized by gastroenterologists [35]. The psychological burden in IBD patients is threefold higher than that in the general population [36]. It is well documented that depression and anxiety may affect more than 25 to 30 percent of individuals with IBD [37, 38]. As expected, the results of the mixed methods in our study indicated that psychological burden is an unavoidable theme for patients with Crohn's disease. These psychological burdens come mainly from personal, family and social pressures. Some posts stated that the disease had caused many impacts on their families and made them vulnerable. These patients lived with low self-esteem, depression and anxiety. Patients with stoma mentioned suicide and death in some posts. A cohort study showed that intestinal surgery and stoma formation are positively associated with subsequent antidepressant medication use [39]. Some people could not support their parents and felt that they were a burden on their family, which caused them to bear a heavy mental burden. In addition, some patients had given up their original work, lifestyle and social contacts, and some had experienced great changes in their lives, such as breakups and divorce. IBD affects women differently than men. Truta’s review summarized the impact of IBD, Crohn's disease and ulcerative colitis on women's health, and found that women with Crohn's disease report worse psychological wellbeing less resilience than men but develop more escape and avoidance strategies to cope with the disease [40]. Our results also support the conclusion. We also found that most women with Crohn's disease were concerned about pregnancy. This is usually an important factor affecting marital happiness and family harmony, as traditional Chinese families need children to carry on the family lineage. IBD frequently affects women of childbearing age and may have implications for pregnancy [41]. Therefore, preconception counseling is pertinent to provide patient education, medication review for risk of teratogenicity and objective disease assessment.

Fortunately, we also found that peer support can play a role in encouragement. Psychological support is not incorporated into the routine care of persons with IBD. Some patients shared their successful treatment experiences, which relieved stress and gave confidence to other patients. Peer support provides a unique perspective of shared experience that can instill hope and assist coping efforts of patients and family [42]. A systematic review found that psychotherapeutic interventions can improve quality of life in patients with IBD and that early intervention after diagnosis leads to better coping strategies and quality of life throughout life [43]. Cumulatively, based on these results, we confirm that psychotherapy is necessary for IBD patients with mental stress.

Diet and nutrition are increasingly becoming matters of interest for Crohn's disease treatment, although they formerly played a marginal role [44]. Decreased dietary intake, nutrient malabsorption and weight loss are some of the nutritional challenges that Crohn's disease patients face [45]. Paradoxically, dietary intake may also trigger the symptoms of Crohn's disease patients [46]. Indeed, a Western-style diet rich in saturated fat and low in fiber has been implicated in the onset of the disease. This leads patients to seek dietary solutions for disease management [47]. However, current dietary recommendations are based largely on low-quality studies. At present, no clear indications of a specific diet are available. However, personalized nutrition is considered a potential way to treat patients with Crohn's disease [44]. One study explored the experience of dietary practices of Chinese patients with IBD and believed that health care professionals should encourage patients to report diet modification and be aware of both personal and environmental barriers to diet modification. [48]. Our study showed that patients with Crohn's disease are confused about their diet. Some posts shared menus and diet experiences with other patients. Vegetables, meat, fruits and beverages were mentioned repeatedly in posts. Malnutrition is detected in approximately 65–75% of patients with Crohn's disease [49]. Enteral nutrition (EN) and parenteral nutrition (PN) are recommended by the guidelines of the European Society of Clinical Nutrition and Metabolism (ESPEN) for malnourished patients [50]. Due to the high cost and side effects of PN, EN is often the first choice for Crohn's disease patients [51], and nasogastric feeding is the main EN method. One study explored the initial factors and experiences of nasogastric feeding by Crohn's disease patients from China, and the results suggested that many patients rejected nasogastric feeding due to fear, image and activity restrictions [52]. Nasogastric feeding is also an important term in our results. As mentioned above, the appearance problem caused by nasal feeding is one of the social pressures of Crohn's disease patients. Given women’s more fragile profile, they tend to be more affected by the effects of the disease than men, so it is necessary to carry out personalized nursing for female patients and improve their quality of life [40].

Weight is one of the nutritional concerns of Crohn's disease patients, who can simply judge their nutritional status by weight changes. However, 15 to 40% of patients with IBD are obese, which may lead to the development of IBD and make colorectal surgery technically challenging [53]. Recently, an integrative review suggested that physical activity can improve quality of life, mental health, sleep quality, fatigue and body weight [54]. In our study, exercise was one of the topics of concern for people with Crohn's disease. Many Crohn's disease patients do not know what and how much exercise will be beneficial. This suggests that the treatment of Crohn's disease requires the participation of nutritionists and exercise scientists to provide a more reasonable plan.

Our results reveal the poor quality of life of patients with Crohn's disease who suffer from intestinal symptoms, systemic symptoms, emotional function, etc. The Inflammatory Bowel Disease Questionnaire (IBDQ) is a valuable disease-specific quality of life questionnaire, that aids in identifying clinical disorientation in patients [55]. The IBDQ has been translated into many languages since its inception [56, 57], and has been used in many ways given its good reliability and validity. It has greatly contributed to evaluating the quality of life of patients and promoting the improvement of IBD diagnosis and treatment strategies. The mainland Chinese IBDQ has proven to be a valid, discriminative, and reliable instrument for assessing health-related quality of life in patients with ulcerative colitis and Crohn's disease in mainland China [57].

COVID-19 has caused a global health crisis, precisely in the country that is the cradle of the COVID-19 infection. In this study, four topics emerged on social media as the ones most discussed by Crohn's disease patients. In the face of this pandemic, patients with Crohn's disease are eager to receive advice on prevention and protection measures. Some researchers have recently provided patients with suggestions on IBD management [58], but at the beginning of the pandemic, there were no suggestion to provide. In addition, patients with Crohn's disease expressed concern about the shortage of medical resources. A study from the Netherlands showed that patients with IBD showed an overall low health-related quality of life during the COVID-19 pandemic, especially the older patients, women and patients who underwent surgical procedures [59]. Many hospitals temporarily closed the biological reagent injection room for the sake of epidemic safety, which led to many patients not being treated on time. Some radical and unprecedented health policies, such as shielding, disrupted patients’treatment and lives. Mass vaccination programs provide the best opportunity for controlling transmission and protecting populations [60], but this is a difficult decision for IBD patients as no confirmed data are presently available regarding COVID-19 vaccines in this population due to their exclusion from the conducted clinical trials [61]. The good news is that some recent studies have provided some clinical evidence [62–64]. Drug use after COVID-19 infection has also become a psychological burden for Crohn's disease patients. A clinical study of 1439 IBD patients from 47 countries showed that combination therapy (TNF antagonist and thiopurine) and thiopurine drugs may increase the risk of severe COVID-19. No significant differences were observed when comparing classes of biological agents [65], although post-acute COVID-19 is characterized by gut viral antigen persistence in IBD [66]. More clinical and basic studies are needed to confirm the problem of drug use after COVID-19 infection.

## Conclusion

Social media is patient-centric and helps us better understand the experiences and perceptions of patients. Our study sheds light on the needs and difficulties of Chinese patients with Crohn's disease. In addition, this study can help medical staff predict the thoughts and concerns that Crohn's disease patients may have during the treatment process, facilitate doctor-patient communication, and assist in the formulation of medical policies.

## Data Availability

All the analysis data were accessed from the Crohn’s disease bar of Bait Tieba Online Website (https://tieba.baidu.com/f?ie=utf-8&kw=%E5%85%8B%E7%BD%97%E6%81%A9).
